# Risks, risk assessment and risk competence in toxicology

**DOI:** 10.3205/000213

**Published:** 2015-07-09

**Authors:** Ralf Stahlmann, Aniko Horvath

**Affiliations:** 1 University Hospital Charité Berlin, Institute for Clinical Pharmacology and Toxicology, Berlin, Germany

**Keywords:** toxicology, fundamental knowledge, dose-effect-relationships, low concentrations, known risks, problem awareness

## Abstract

Understanding the toxic effects of xenobiotics requires sound knowledge of physiology and biochemistry. The often described lack of understanding pharmacology/toxicology is therefore primarily caused by the general absence of the necessary fundamental knowledge. Since toxic effects depend on exposure (or dosage) assessing the risks arising from toxic substances also requires quantitative reasoning. Typically public discussions nearly always neglect quantitative aspects and laypersons tend to disregard dose-effect-relationships. One of the main reasons for such disregard is the fact that exposures often occur at extremely low concentrations that can only be perceived intellectually but not by the human senses. However, thresholds in the low exposure range are often scientifically disputed. At the same time, ignorance towards known dangers is wide-spread. Thus, enhancing the risk competence of laypersons will have to be initially restricted to increasing the awareness of existing problems.

## Zusammenfassung

Das Verständnis der toxischen Wirkungen von Stoffen setzt ein gutes Verständnis der Physiologie bzw. Biochemie voraus. Das häufig beschriebene mangelnde Verständnis in der Bevölkerung für die Pharmakologie und Toxikologie beruht deshalb zu einem großen Teil auf den mangelnden Kenntnissen der erforderlichen Grundlagen. Toxische Wirkungen sind dosisabhängig, die Einschätzung von Risiken durch toxische Stoffe setzt also quantitatives Denken voraus. Da öffentliche Diskussionen aber fast immer die quantitativen Aspekte weglassen, werden von Laien Dosis-Wirkung-Beziehungen kaum beachtet. Ein wesentlicher Grund liegt auch in der Tatsache, dass die Expositionen in extrem niedrigen Konzentrationen erfolgen, diese Mengen aber nicht mit den menschlichen Sinnen erfasst werden können. Sie sind nur intellektuell nachvollziehbar. Häufig sind die Grenzwerte im Niedrigexpositionsbereich allerdings auch wissenschaftlich umstritten. Gleichzeitig besteht eine weit verbreitete Ignoranz gegenüber bekannten Gefahren.

Eine Stärkung der Risikokompetenz bei Laien wird sich deshalb zunächst darauf beschränken müssen, für die vorhandenen Probleme ein stärkeres Bewusstsein zu schaffen.

## Introduction

Possible toxicological risks are frequently discussed in daily life. Such risks often dominate media headlines, subsequently achieving wide dissemination. Fears generated by these risks in large sections of the population are often exploited for other purposes. The media are interested in the dissemination of such risks because of the associated increase in attention, and political issues are often ‘spiced up’ with alleged toxicological risks in order to assert somebody’s interest. This article starts with a few comments on the identification and assessment of toxicological risk to show why so many public debates lack the necessary risk competence. A more detailed report on toxicological risk assessment is mentioned in the references [1].

## Poor understanding of pharmacological and toxicological effects

The main focus of pharmacology and toxicology is the desired and undesired or adverse effects of chemical substances on living organisms. One important task in this field is the identification of the basic mechanisms of action that means, the interaction between the chemical substance and the biological structures relevant for the effect at the molecular level. Understanding the toxicological effects of substances thus requires sound knowledge of physiology and biochemistry to answer the question about the mode of action of a poison or drug.

The often described people’s lack of understanding pharmacology and toxicology is probably mainly caused by the general non-existence of the necessary basic knowledge. Both pharmacological and toxicological facts are often poorly understood. For instance, the development and use of immunosuppressive agents is a key requirement of transplantation medicine. Yet, the ordinary person seems to comprehend surgical interventions better than the mode of action of immunosuppressants which requires at least basic knowledge of the function of the immune system. As a consequence, medical advances in this field become more commonly associated with surgery than with pharmacology and are not viewed as interdisciplinary research success.

## The importance of quantitative reasoning in toxicology

Pharmacological and toxicological effects are dose-dependent. The risk of a toxicological effect increases with increasing exposure. Assessing the risk posed by toxicological substances also necessitates quantitative reasoning. This concept was already described by Paracelsus in his famous quote on the dose dependence of effects about 500 years ago. All substances are ‘toxic’ in high quantities and ‘non-toxic’ in lower quantities, but the public discussions show that quantitative aspects are mostly ignored. One of the main reasons for the avoidance of statements on quantities and concentrations, for instance in the media, probably is that exposure mostly occurs at extremely low concentrations (in the range of micrograms, nanograms and even picograms). Such low concentrations can only be perceived intellectually but not by the human senses. Because these concentrations are rather insignificant in daily life, they are probably disregarded by most laypeople.

A few decades ago, there were no public discussions about increased concentrations of ‘dioxins’ and other contaminants in food products. Only a few consumers are aware of the fact that, for instance, dioxins are ubiquitous substances in our environment and in fatty foods. Only since the 1980s, however, has the analytical determination of such substances been possible and economically justifiable as a routine procedure. One picogram per gram or 1 ppt (parts per trillion) specifies a ratio of 1:10^12^. Transferred to distances, this ratio would describe less than the thickness of a hair (0.04 mm) in proportion to the circumference of the earth measuring approximately 40,000 km. The following example draws an even more visual picture: Today's techniques would allow the analysis of one sugar cube with an average weight of 5 gram, dissolved and equally distributed in Lake Constance with a surface of about 535 square kilometres and a water volume of over 48 billion cubic metres [2].

## The determination of thresholds and the associated contemporary discussion

The terms ‘hazard’ and ‘risk’ relate to completely different circumstances. Hazard, which should be understood in a qualitative sense, can be used for substances with a toxic potential. In contrast, risk should be understood in a quantitative sense because this term describes the incidence of an adverse effect at a certain level of exposure, hence a definable quantity. This definition is based on human data; thus, it retrospectively refers to a situation in which the damage to people has already occured. Prevention is not possible this way.

In toxicology, two different approaches have to be distinguished: medical risk assessment and preventive risk minimisation. Both approaches ultimately help to reduce or prevent risk to human health. In risk assessment, dose-effect-relationships and definite incidence rates may be stated. In prevention, certain thresholds are estimated below which health risks can be excluded or considered ‘acceptable’. Prevention calculations are mostly based on experimental animal data and extrapolated to humans; thus, such calculations often include (un)certainty factors which are based on more or less secured assumptions.

## Risk assessment on the basis of human data

Experimental animal data are very important with regard to the tolerability of medicines because they usually reveal the potential for certain adverse effects. Such effects may then be specifically monitored in clinical studies. In the advanced stage of drug testing, prospective randomised and, if possible, double-blind clinical studies have become the basis for identifying desired as well as adverse effects. Rarely occurring serious adverse effects cannot be detected in view of the limited number of patients participating in phase I, II and III studies. For this reason, drugs are sometimes withdrawn from the market, or warnings about possible risks are increased.

Nowadays, more and more ‘pregnancy registries’ for drugs with teratogenic potential are established because of the risk of foetal malformation. Women are prospectively registered in case of unintentional drug intake in the early stages of pregnancy, thus during the first few weeks when women often do not yet know about their pregnancy. An example in this respect is Topiramate, a relatively new antiepileptic drug that is also used for migraine prophylaxis. The teratogenic effect of many antiepileptics in humans has been known for decades. The teratogenic potential of Topiramate has been shown in preclinical tests in mice, rats and rabbits. However, these effects occur at very different doses which are well above the doses used for humans. Furthermore, such findings are difficult to interpret because of simultaneously occurring toxic effects in the dams (maternal toxicity). Initially it remained unclear if these experimental findings were also important for humans (Table 1 (Tab. 1)). In the years after the approval of Topiramate, the North American Antiepileptic Drug Pregnancy Registry (NAAED) showed an increasing number of foetal malformations. After prenatal exposure to Topiramate, the incidence of cleft lip and palate in children was 1.2% in comparison to 0.4% for other antiepileptic drugs; the normal rate of this malformation in the general public is 0.1% [3]. The warnings regarding the intake of this drug during pregnancy were subsequently increased. Careful risk-benefit evaluations in consideration of the indication are required for each individual patient if the drug is prescribed to women of childbearing age. 

## The NOAEL safety factor concept

Dose-effect-relationships in the low-dose range cannot be distinguished in experiments. For instance, determining an effect incidence of 1/100 or 1/1,000 with the required safety margin would require the examination of an unjustifiably high number of animals. Serious toxic effects affecting one in a hundred or one in a thousand people are not acceptable in most circumstances. For this reason, comparatively high doses are used in animal experiments, and the results have to be extrapolated from high to low exposure and from one species to another. The higher the gap between the experimentally investigated doses and the exposures to be reviewed, the higher is the uncertainty in extrapolation. It should be taken into consideration that high doses are often marked by several – and often interacting – effects, resulting in complex dose-effect-relationships. Because the course of a dose-effect-relationship curve in the low-dose range is unknown, a no observed adverse effect level (NOAEL) is initially determined, hence a dose for which no toxic effect can be found in the experiments. A safe range of exposure (for instance, acceptable daily intake – ADI) is then defined by means of an (un)safety factor. The applied factors are usually round numbers, such as 100, 300 or 1,000, which shows that these factors are not based on science but serve an administrative and political purpose. In the context of health politics, this concept appears rather appropriate because of often missing data on the mode of action of a substance and other knowledge gaps.

Thresholds defined by different institutions or the legislature have both a regulating and a communicative function to prevent health risks and damage to the ecosystem. Risk analyses are mainly based on toxicological and epidemiological data. Four different phases have to be differentiated: Identification of potential hazard, analysis of the dose-effect relationship, analysis of the exposure and identification of populations at risk (Table 2 (Tab. 2)). Because of the general dose dependency of effects due to xenobiotics, thresholds have to be established for each substance, by which exposure irrelevant to health can be distinguished from exposure relevant to health. Thresholds in the low exposure range are often scientifically disputed. This range is marked by speculations because meaningful prospective studies, such as in drug development, are ethically not justifiable in most cases. However, retrospectively collected data often contain confounding elements, and the significance of such data is often somewhat overrated. Relative risks found in such studies have to be differentiated from risks found in the context of risk assessments that constitute incidences. A relative risk is the ratio of an exposed group and a control group.

A cause-effect-relationship cannot be proven by means of an epidemiological study. The results of such studies have to be confirmed in additional independently conducted studies, which often yield opposite results. A layperson, who often cannot follow the scientific argumentation in detail, usually will only remember the negative finding which is often more intensively communicated in public. The problematic nature of epidemiological studies was clearly and comprehensibly presented in a review some years ago [4]. Even if the strengths and limitations of a study have been carefully weighted scientifically, only the problematic results make headlines and become public knowledge. If a slightly increased relative risk of 1.8 has been found for the association between a substance and an effect – for instance, in cancer diseases –, it would be absurd to present this figure as evidence for an 80% increased risk. Most epidemiologists advocate accepting only a relative risk of 3 or 4 as a serious result. Less distinct increases are often used for creating news under the headline ‘anxiety of the week’ [4].

## The example of aluminium

As an ubiquitously disributed substance, aluminium shall serve as an example of the insufficient amount of data available. The toxicological risks posed by exposure to aluminium have been the subject of public debate for many years. Very low thresholds apply for aluminium in the case of actual exposure through nutrition, but these thresholds are clearly exceeded in medical interventions. The thresholds for food products are exceeded many times after the intake of over-the-counter antacids or the administration of vaccines. Aluminium and its compounds have a significantly lower toxic potential than other metals. Nevertheless, cancer diseases as well as neurological diseases such as Alzheimer’s continue to be associated with aluminium exposure. In view of the various exposures to aluminium, some consumers have developed a phobia to this metal. 

Current disturbing news reports concern the exposure to antiperspirants and comments by governmental authorities on the lack of reliable data regarding the absorption of substances through the skin. Only a very small portion of aluminium in food products is reabsorbed, usually about 0.1% to 0.3%. However, the bioavailability of aluminium is significantly increased in the presence of citric acid, and its kinetic behaviour seems to differ between species which makes an extrapolation of the experimental findings in animals to humans difficult. Calculating the maximum acceptable human exposure thus involves a degree of uncertainty. Because aluminium is rapidly excreted via the kidneys, patients with impaired kidney function have a higher risk of adverse effects. Dialysis fluids with above normal aluminium content are known to cause serious neurological complications in patients requiring dialysis.

With regard to food exposure, a threshold value of 1 mg aluminium per kilogramme body weight per week (TWI, tolerable weekly intake) has been established by the European Food Safety Authority (EFSA). Taking a resorption rate of 0.1% into consideration, an amount of approximately 10 µg per day may be acceptable for adults of average body weight. If and how much aluminium is absorbed from cosmetics such as antiperspirants is not known, but rudimentary data indicate that the amount of aluminium absorbed through the skin is much lower than the amount absorbed through the gastrointestinal tract. Further tests on this issue are considered absolutely essential. Valid data on human exposure are an essential prerequisite for sound risk assessment. So far, epidemiological studies have not yielded any indication that exposure to aluminium through the use of cosmetic products may lead to neurological diseases or cancer diseases. Chronic feeding studies with mice have shown that extremely high doses of aluminium even reduced the incidence of some types of cancer.

## Risk perception and intuitive risk assessment

The perception of dangers and risks is influenced by several factors. Different types of risk exist, and risks are often overestimated or underestimated. When asked about their risk of disease, many people view their risk as lower than that of their peers. Such underestimation of one’s own risk is termed ‘unrealistic optimism’ or ‘optimistic misconception’. In contrast, pessimistic misconceptions, that means, overestimating one’s own risk compared to the risk of one’s peers, is very rare [5], [6], [7].

Risk perception and risk assessment often largely differ between laypeople and experts. Such perceptual differences are often caused by demographic reasons [8] and the associated knowledge gap. Risk perception may be increased by different knowledge sources and media reporting. The risk assessment approaches of laypeople tend to be more emotional, are more often based on opinions than on knowledge, and often ignore probabilities. Social perceptual differences also depend on media reporting, the ordinariness and the frequency of the risk (car accident or plane crash) as well as the degree of horror (terrorist attack of 9/11). Several analyses have shown that risks taken voluntarily are often viewed as controllable. Uncontrollable risks are not only considered unintentional, terrible and horrible, but as risks with fatal consequences that will also endanger future generations and increase in risk intensity [9].

Studies have shown that laypeople hardly ever differentiate between the different levels of toxicity and often disregard dose-effect-relationships. A study comparing toxicologists and laypeople with regard to their assessment of risks due to foreign matters showed that most laypersons regard the mere contact with a toxic substance as a health risk. Most laypeople also believe that results obtained in animal studies can be simply transferred to humans. However, hardly anybody who has never addressed this problem in detail would know that the comparison of selected species necessitates in-depth knowledge on the kinetic behaviour and metabolism of humans and animals. The risk assessment of many laypeople – in contrast to that of most toxicologists – depends on the nature of a substance, that means, whether a substance is of natural or synthetic origin. Most laypeople consider chemically defined substances of natural origin less dangerous as synthetically produced compounds, which is in line with the myth ‘nature is benevolent, and natural means safe’ [10], [9].

## Pyrrolizidine alkaloids – toxicologically relevant natural substances

In this context, a statement on ‘pyrrolizidine alkaloids in herbal and non-herbal teas’ was published by the German Federal Institute for Risk Assessment in 2013. Pyrrolizidine alkaloids are secondary plant ingredients generated by a wide variety of the world’s plant species for protection against herbivores. These alkaloids are particularly found in plants belonging to the legume family and the family of Asteracee. Even small quantities of these alkaloids may cause acute poisoning in humans; even more critical is their ability to modify deoxyribonucleic acid (DNA) which may lead to gentoxic and carcinogenic effects, possibly already at a low level of exposure.

Already in 1988 did the World Health Organisation (WHO) inform about the cumulative nature of the toxic effects of pyrrolizidine alkaloids and the health risk of even low chronic exposure. Long-term effects in humans include cirrhosis of the liver and the development of tumours. So far, no valid clinical studies on humans are available investigating dose-effect-relationships in the long term. One reason for the unavailability of such studies is the lack of valid and routinely applicable analytical methods.

Only about 20 of the more than 500 chemically related, naturally occurring alkaloids are available as a reference substance for analysis. Measuring the concentration of individual substances does not show all aspects of exposure. In view of the high genotoxic potency, even the lowest concentrations are critical, so that analyses have to be done in the lower thresholds of the nanogramme per kilogramme range. Several methods have been described but none of the available procedures has yet been established as an official control method. The ordinary person will hardly be able to understand what performance requirements have to be met regarding sample purification and sample concentration as well as with regard to the detection of analytes. The method – developed by the German Federal Institute for Risk Assessment to determine alkaloids in teas – was first validated in an internal process and is now being validated in an international collaborative ring trial with the aim to standardise this method [11].

The German Federal Institute for Risk Assessment uses an approach termed ‘margin of exposure’ (MOE) for evaluating a possible long-term establishment of this method. This approach is used internationally to assess the potential risk of gentoxic and carcinogenic acting substances. The MOE approach constitutes a method for risk description originating from exposure to carcinogenic or gentoxic substances in food products. The MOE value is the ratio of two factors in a particular population: the smallest dose for which a marginal but measurable adverse effect can be observed and the level of exposure to the respective substance. The higher the MOE value, the lower is the potential health risk for the consumer. Based on the results obtained in animal studies, genotoxic effects with MOE values of 10,000 or higher are assumed to bear small risk. According to the results of the project managed by the German Federal Institute for Risk Assessment, the MOE value for the intake of pyrrolizidine alkaloids would be significantly lower than 10,000 for both adults and children if such alkaloids are consumed frequently.

The German Federal Institute for Risk Assessment came to the conclusion that short-term ingestion is unlikely to present any acute health risk for adults or children. Long-term ingestion, however, may pose a risk to children as well as to pregnant and breastfeeding women. Consequently, it is recommended to offer children a variety of different teas as well as other beverages.

## Ignorance towards real dangers

The fact that a poorly functioning stove or fireplace can be dangerous to life is well known. Nevertheless, many people die as a result of carbon monoxide poisoning in Germany every year. Attention has been frequently drawn to the danger of having a barbecue in a closed room because burning charcoal releases large quantities of toxic gases, particularly carbon monoxide (CO), which may result in death. In the framework of a test series conducted by the German Federal Institute for Risk Assessment and the German Federal Institute for Materials Research and Testing, 800 grams of charcoal were burnt in a cloud chamber sized 19 cubic metres. After two hours, CO concentrations of more than 3,000 ppm (parts per million) were measured. Any person inhaling such an indoor air concentration would be unconscious after only a few minutes. The danger emanating from glowing charcoal and the associated risks are well known; nevertheless, products such as indoor barbecues or cooking pots fired with charcoal are regularly put onto the market. 

Other studies have shown that smoke detectors do not offer any protection from carbon monoxide poisoning. Smoke alarms are able to detect finest articles in the air which develop when a fire breaks out. Glowing charcoal, however, almost exclusively releases invisible fumes that cannot be detected by optical smoke detectors [12]. 

Appropriate communications on this topic made by the German Federal Institute for Risk Assessment over the past few years were given less public attention than reports on slightly increased dioxin concentrations in food products. This institute has been undertaking major efforts to increase risk communication; under the header ‘risk communication’, the institute’s website provides more than 200 presentations, press releases, conference transcripts and other information material.

## Enhancing risk competence

Risk competence presupposes that a person is capable to adequately identify and assess a risk as well as to subsequently deal with the risk in a rational manner. Such capability would also include a basic understanding of statistical correlations. As already mentioned, understanding toxic effects necessitates the basic knowledge of scientific subjects such as biology and chemistry; yet, laypeople often lack such knowledge. Despite increased efforts to inform the public on interrelations, real dangers are often ignored and other dangers overestimated. Without any better education in schools and universities, such discernible efforts to improve the situation will also have only limited success in future. The Max Planck Institute of Education Science in Berlin has already addressed this problem by means of a pilot project in schools to help children acquire important basic skills [13]. Such initiatives should be developed further.

The employment of reliable knowledge disseminators, such as physicians, pharmacists, teachers and educators, should be further extended in order to give people an understanding of possible health risks as quickly and directly as possible. Pharmaceutical personnel in public pharmacies, for instance, are competent contact persons within easy reach. When patients present a prescription or buy an over-the-counter drug, pharmacists will inform them about possible risks and explain medication administration information in a personal and direct conversation – a service denied to customers of internet pharmacies.

Unfortunately, many people are unable to take drugs according to the instructions printed on the patient information leaflets which come with the medications [14]. Such patient information is highly relevant because many patients do not really know why a drug is prescribed and how they should take the medication. In the U.S. states Illinois, Louisiana and Michigan, a trial was conducted in out-patient health care centres in urban areas with a high proportion of socioeconomically disadvantaged people. 395 English-speaking patients aged 18 years and above were asked if patients understood the information provided on patient information leaflets which come with the medications. The average age of the respondents was 45 years (ranging from 18 to 85 years), two thirds were women, 28% did not have a high school degree, and the reading comprehension of 48% of respondents corresponded to that of a 14-year old person. Respondents were asked to give the dosage, time(s) of day of the intake and duration of therapy of two different antibiotics, one antihypertensive drug, one cough expectorant and one diuretic drug. The results were rather depressing: 19% of the responses were wrong, and 46% of the respondents had not understood every instruction of at least one patient information leaflet. The most common problem was the combination of different numerical instructions, such as one pill two times a day for seven days. To what extent these results can be transferred to Germany remains unclear, but the preparation of patient information and package leaflets in an easily-comprehensible that can be understood by laypeople should be given top priority [14].

A further important issue is the continuous development of educational and training programmes for physicians. In 2012, Wegwarth and Gigerenzer published the results of a study including approximately 300 general physicians in the United States. The majority of physicians was unable to differentiate between relevant and irrelevant data of a statistics on cancer screening and did not even detect misleading information. German physicians also had this problem and did not understand every side effect described in patient information leaflets [15], [16]. Different information channels, for instance, brochures, newspapers, internet and television, need to be used for informing the public about possible hazards and risks. The main goal should be to bring excessive alarmism on the one hand and disinterest on the other hand to a realistic and appropriate level.

## Summary

**Figure 1:**
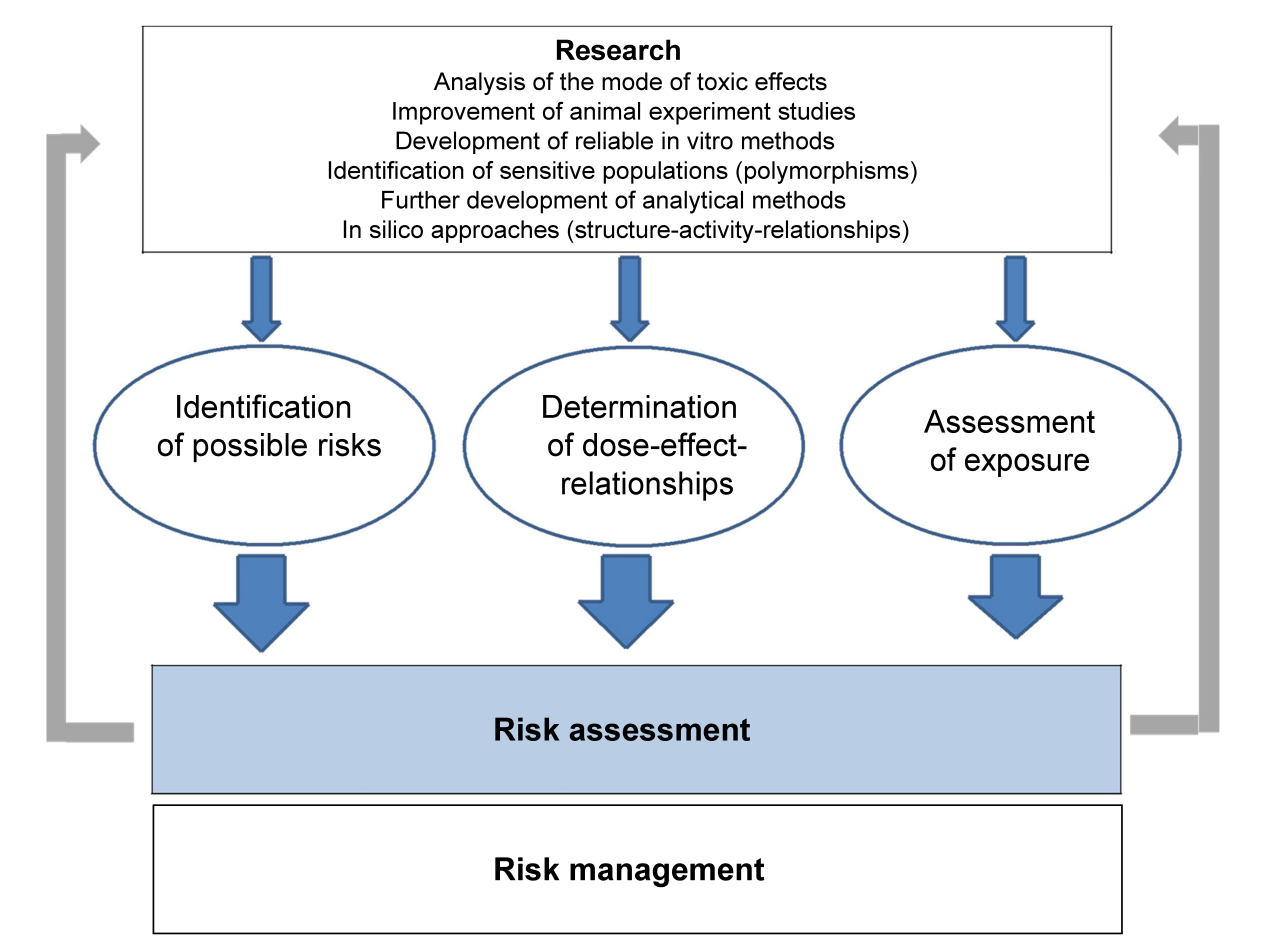
The assessment of toxicological risks is a complex, multistage process followed by risk management measures. New stimuli provided by research are based on the further development of existing methods as well as on the establishment of new methods, such as ‘in silico toxicology’, computer-aided methods of structure-activity relationships and other approaches. Detailed knowledge of the mode of action (toxicodynamics) and the behaviour of xenobiotics in the organism (toxicokinetics) are the basis of solid risk assessment. On the other hand, research also benefits from knowledge gained from processes of risk assessment because of the identification of knowledge gaps and future research necessities.

The rather complex determination and assessment of toxicological risks requires multistage concepts (Figure 1 (Fig. 1)). The starting point is mostly results obtained in animal experiments from which potential risks are derived. Human data are essential for the determination of risks regarding a defined exposure to xenobiotics. Risks derived from randomised prospective clinical studies, for example in the context of drug development, show causal relations. If such risks are derived from data obtained in retrospective studies, it should be taken into consideration that such data often cause ‘false alarms’. Such slightly increased relative risks may not be confused with risks derived from prospective clinical trials. Retrospective approaches may only be used for the determination of correlations. Because of the complexity of the matter, enhancing the risk competence of laypersons will initially have to be restricted to outlining and propagating the problem of possible misinterpretations and their causes.

## Notes

### Competing interests

The authors declare that they have no competing interests.

## Figures and Tables

**Table 1:**
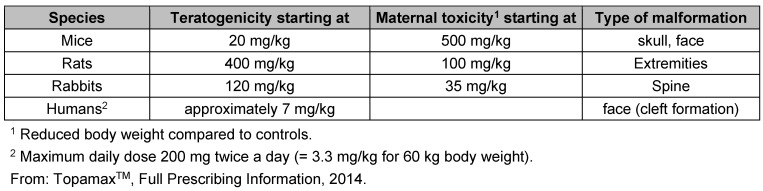
Teratogenic effects of the antiepileptic drug Topiramate in humans and various animals species

**Table 2:**
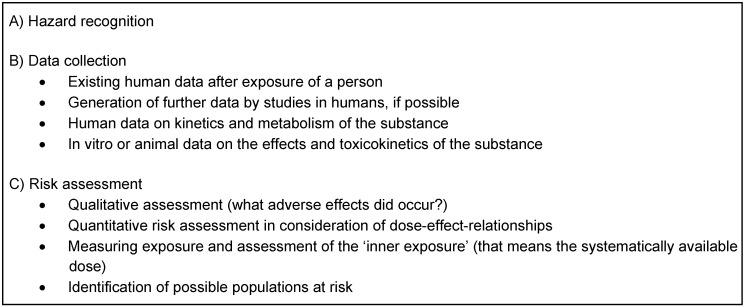
Procedure of toxicological risk assessment
